# Evaluation of patients with wild mushroom poisoning in the emergency department

**DOI:** 10.1186/cc14592

**Published:** 2015-03-16

**Authors:** M Altuntas, L Duran, T Yardan, H Akdemir

**Affiliations:** 1 Ondokuz Mayis University School of Medicine, Samsun, Turkey

## Introduction

Wild mushroom poisoning (MP) is an important medical emergency that may have bad clinical outcome. We aimed to evaluate the clinical and laboratory features of patients with wild MP admitted to our emergency department in the Central Black Sea Region and to inform the emergency department physicians about early diagnosis and management of wild MP in the light of obtained data.

## Methods

This study was designed retrospectively by examining files of the patients with wild MP who were admitted to Ondokuz Mayis University Emergency Department, between January 2008 and December 2012. Patients older than 18 years were included in the study. Patients were evaluated according to gender, age, location, duration between mushroom intake and the start of clinical symptoms, time of application to hospital, clinical features and findings and treatment method. The number of patients has been compared with the regional distribution of population, monthly temperature and average annual rainfall.

## Results

A total of 420 patients poisoned by wild mushrooms were studied. The male/female ratio was 1/1.5. The age of patients changed from 18 to 92 and mean age was 46 years. MP constituted 13.3% of all intoxication cases. The time when the first symptom occurred after mushroom intake was a mean 2 (0.17 to 2.15) hours. Of the patients, 47.6% lived in villages, 38.6% in towns and 13.8% in city centers. Admissions were mostly made in autumn, with 57.6%. Eighty-six percent of intoxications happened because of wild mushrooms collected in nature. The most frequent symptoms were nausea (93.8%), and vomiting (87.1%). Increase in liver function tests in 47 patients was observed. Two of these patients died while 10 patients were transferred to further centers for liver transplantation. The remaining patients were discharged from the hospital.

## Conclusion

Wild MP can cause bad clinical outcome. The public should be informed about the probable hazards of wild mushroom ingestion because collection and consumption of wild mushrooms from nature is common. Public health units should take protective precautions against wild MP. Education of health personals regarding MP will lead to successful results in patient management.

